# Participation and Equity in Long-Term Care: Systemic and Societal Perspectives

**DOI:** 10.1093/geroni/igaf122.2693

**Published:** 2025-12-31

**Authors:** Sandra Staudacher, Nora Peduzzi, Séverine Soiron, Katja Jungo, Norina Reiffer, Alexandra Staehli, Andrea Kaiser-Grolimund, Franziska Zuniga

**Affiliations:** University of Basel, Basel, Basel-Stadt, Switzerland; University of Basel, Basel, Basel-Stadt, Switzerland; University of Basel, Basel, Basel-Stadt, Switzerland; University of Basel, Basel, Basel-Stadt, Switzerland; University of Basel, Basel, Basel-Stadt, Switzerland; University of Basel, Basel, Basel-Stadt, Switzerland; University of Basel, Basel, Basel-Stadt, Switzerland; University of Basel, Basel, Basel-Stadt, Switzerland

## Abstract

As global population aging increases, so does the demand for long-term care (LTC), particularly in care homes, assisted living, and home-based care. Many older people hesitate to enter care homes, fearing a loss of autonomy and participation in society. This presentation investigates how participation of older people in LTC in Switzerland is understood, discussed, and practiced, and analyzes potential inequities within the LTC system. As part of the Swiss National Science Foundation-funded EPICENTRE-PARTICIPATIO project, we conducted an ethnographic analysis of participation across the LTC system in Switzerland, including care homes, home-based care, assisted living, public administration, associations, and civil society. Fieldwork consisted of observations, interviews, and document analysis to examine how decisions about and with older people in LTC are made. The study reveals how different actors in the LTC system and society conceptualize participation, and where it is practiced, missed, or resisted. Findings highlight systematic inequalities in participation between social groups, shaped by factors such as cognitive or physical impairment, gender, and migration background. Socio-cultural, political, and economic contexts significantly influence participation. Addressing these disparities requires a holistic approach, including a differentiated understanding of LTC populations and consideration of external factors like policymaking and financial constraints. Applying the concept of equity provides a useful lens for LTC research on participation, emphasizing the need to address inequalities both within LTC and compared to other social groups.

